# Impact of dental anxiety on dental caries and salivary alkaline phosphatase in children across different nutritional statuses

**DOI:** 10.25122/jml-2023-0085

**Published:** 2023-10

**Authors:** Raya Rashid Aldafaai, Zainab Jafar, Yamama Al-Rubbaey

**Affiliations:** 1 Department of Clinical Sciences, College of Dentistry, Ibn Sina University of Medical and Pharmaceutical Sciences, Baghdad, Iraq; 2 Department of Pedodontics and Preventive Dentistry, College of Dentistry, University of Baghdad, Iraq

**Keywords:** dental anxiety, dental caries, salivary alkaline phosphatase

## Abstract

Dental anxiety is a familiar problem among children, which may affect their oral health. This cross-sectional study aimed to evaluate dental anxiety during the first dental examination in relation to dental caries and salivary alkaline phosphatase, taking into account the nutritional status of children. Eighty-two children (45 boys and 37 girls), aged six to eight years old (average 6.96), were recruited from public clinics in Baghdad city. Participants were divided into the anxious and the non-anxious groups according to physiological measures (oxygen saturation and heart rate) before the first dental treatment. The weight and height of each child were measured to determine the nutritional status (normal weight, overweight, and obese). Dental caries (prevalence and severity) in children with different nutritional statuses were recorded using dmf, DMF, and the guidelines outlined by Manjie *et al*. Saliva was collected to analyze salivary alkaline phosphatase. No statistically significant differences were observed between anxious and non-anxious children in means of dmf and DMF indices (teeth and surfaces) in primary and permanent teeth (p>0.05) in the three nutritional status groups. However, non-anxious children exhibited a higher mean of d1 (initial enamel caries) compared to anxious children, with statistical significance (p<0.05) observed only in normal-weight children. Salivary alkaline phosphatase was not significantly different between the two groups. In conclusion, dental anxiety did not appear to significantly impact caries development in primary teeth or salivary alkaline phosphatase levels in children with varying nutritional statuses. Anxiety reduced the initial enamel caries in normal-weight children.

## INTRODUCTION

Dental anxiety is a negative and excessive emotional status associated with dental treatment among dental patients. Various methods are employed to assess dental anxiety, including behavioral assessment, psychometric questionnaires, and physiological response analysis [1]. In children, dental anxiety may be influenced by a range of psychosocial factors, such as a child's personality, prior medical experiences, parental dental anxiety, and socio-economic factors [2]. The relationship between dental anxiety and dental caries is controversial. While some studies have reported associations between dental anxiety and caries prevalence [3-5], others, such as Taani *et al*., have found no such correlation [6]. In addition, the relationship between dental caries and nutritional status among children was studied previously due to interrelated factors like psychosocial, diet, genetics, and education. The literature is rich in studies that evaluated the associations between these factors in different populations; however, the results are still controversial. Some studies have linked dental caries with obesity or underweight in children [7-8]. Additionally, research has suggested that dental anxiety and a child's first dental appointment can impact salivary biomarkers, including cortisol [9], potentially affecting the levels of alkaline phosphatase enzyme. The alkaline phosphatase (AlP) enzyme in saliva is important for re-mineralization and is most active at alkaline pH [9, 10]. Differences in salivary enzymes like alkaline phosphatase cause changes in phosphate levels, which could lead to differences in dental caries occurrence [10, 11]. However, no evidence was found on the relation between salivary AlP and dental anxiety. On the other hand, higher levels of alkaline phosphatase enzyme were connected to the occurrence of obesity [12]. As there were no previous studies on the effect of dental anxiety in children with different nutritional status on dental caries and salivary alkaline phosphatase, this study was conducted. The null hypothesis of the study was that dental anxiety has no effect on caries occurrence and salivary alkaline phosphatase in children with different nutritional statuses. The objectives of this study were to determine dental anxiety using physiological measurements in children, quantifying the occurrence and severity of dental caries using the d1, d2, d3, d4 indices for deciduous teeth and the D1, D2, D3, D4 for permanent teeth, and salivary alkaline phosphatase.

## MATERIAL AND METHODS

### Study design and participants

This cross-sectional study enrolled children aged six to eight years from randomly selected public clinics in the Karkh sector of Baghdad city. The sample size was determined using G*Power software, with a study power of 80% and an alpha probability error set at 0.05. The statistical test employed was the two Independent Sample T-test. A pilot study involving ten subjects was conducted to compare the means of the decayed, missing, and filled permanent teeth or surfaces (DMFS) between anxious and non-anxious groups. The mean ± standard deviation (SD) for the DMFS in the pilot study were 1.1±0.213 and 0.955±0.212 for the anxious and non-anxious groups, respectively. Based on a medium effect size (Cohen's D effect size of 0.682), the required sample size was calculated to be 35 subjects for each group (70 subjects). Accounting for a 10% error rate, the final sample size was determined to be 82 subjects, which included 10 participants from the pilot study. Inclusion criteria involved children with no systemic diseases and no previous dental visits. Exclusion criteria encompassed children with a history of medical problems, mental disorders, or prior dental clinic visits.

### Dental examination

Oral examinations were conducted following the 1987 criteria set by the World Health Organization (WHO) to assess dental caries in primary teeth (dmft, dmfs) and permanent teeth decayed missing and filled surfaces (DMFT, DMFS). Furthermore, the severity of dental caries was assessed following the guidelines outlined by Manjie *et al*. in 1989, encompassing the evaluation of d1-4 for primary teeth and D1-4 for permanent teeth [13]. The oral examination was performed by a specialized dentist.

### Nutritional assessment

Children’s weight was measured using a digital weight scale (China). The scale was calibrated after weighing ten children, using a known weight of ten kilograms to ensure accuracy. Height measurements were taken using a stadiometer (China), with precision to the nearest 0.5 centimeter. Nutritional status was estimated according to the Centers for Disease Control and Prevention (CDC) Growth Charts, considering gender-specific BMI-for-age percentiles. Subsequently, the children were categorized into three groups based on their nutritional status: normal weight, overweight, and obese.

### Assessment of dental anxiety

Dental anxiety was assessed through physiological measures, including oxygen saturation and heart rate, using a pulse oximeter (Biolight, model: M70, China) while the children were in the waiting room. These measurements were also repeated just before the dental treatment in the dental chair. Children with values observed outside the normal range for oxygen saturation (97-99) and heart rate (65-110) were categorized into the anxious group, while those with values within the normal range were considered non-anxious [14].

### Saliva collection

Unstimulated salivary samples (5 ml) were collected between 10 a.m. and 11 a.m. to account for circadian variation. Saliva collection and storage are demonstrated below:

- Patients were instructed not to eat or drink (except water) for one hour before saliva collection.
- A one-minute pre-sampling period was observed for relaxation.
- Patients were seated in a relaxed position in an ordinary chair.
- Saliva samples containing blood were discarded if chemical analyses of saliva were planned.
- Saliva was collected in a plain tube and centrifuged for 10 minutes at 3,000 xg.

The centrifuged supernatant liquid was stored in a deep freeze at -20ºC until the biochemical analysis for detecting alkaline phosphatase [15]. The salivary samples were analyzed in a biochemical laboratory to determine the activity of salivary alkaline phosphatase (AlP) spectrophotometrically using a kit from Human Company, Germany, following the recommendations of the German Clinical Chemistry Association.

### Statistical analysis

Data were analyzed using the Statistical Package for the Social Sciences (SPSS) program, version 23, with a significance level set at less than 0.05 (p<0.05). Shapiro-Wilks test confirmed normal data distribution across all subgroups (p>0.05). Mean values, standard deviations (SD), and independent sample t-tests were utilized to compare the mean values between the anxious and non-anxious groups (independent variables).

## RESULTS

In the current study, 82 participants were included, consisting of 45 boys (54.9%) and 37 girls (45.1%) with an average age of 6.96 years. The participants were categorized into two groups: 40 anxious children and 42 non-anxious children, based on oxygen saturation measurements and heart rate. The mean heart rate values showed a significant difference between the two groups, whereas the difference in mean oxygen saturation values was not statistically significant (Table 1).

**Table 1:** Heart rate and oxygen saturation among anxiety groups

Variables	Anxiety						T value	p-value
	Anxious group			Non-anxious group				
	N	Mean	±SE	N	Mean	±SE		
Oxygen saturation	40	97.8	0.57	42	96.9	0.58	1.12	0.26
Heart rate	40	110.5	1.9	42	80.1	1.02	13.855	0.000*

* Significant difference (p<0.05)

The prevalence of permanent teeth caries across anxiety groups was assessed according to nutritional status (Table 2). No statistically significant differences were observed between anxious and non-anxious children in mean values of DMFS and DMFT and their components.

**Table 2:** Prevalence of permanent teeth caries among anxiety groups based on nutritional status

Nutritional status	Dental caries	Anxiety						T value	p-value
		Anxious			Non-anxious				
		N	Mean	±SE	N	Mean	±SE		
Normal weight	DS	16	0.500	0.224	20	1.158	0.392	1.387	0.175
	MS	16	0.000	0.000	20	0.000	0.000	. . . .	. . . .
	FS	16	0.000	0.000	20	0.000	0.000	. . . .	. . . .
	DMFS	16	0.500	0.224	20	1.100	0.376	1.286	0.207
	DMFT	16	0.438	0.182	20	0.750	0.260	0.936	0.356
Overweight	DS	8	0.625	0.625	15	0.800	0.380	0.253	0.802
	MS	8	0.000	0.000	15	0.000	0.000	. . . .	. . . .
	FS	8	0.000	0.000	15	0.000	0.000	. . . .	. . . .
	DMFS	8	0.625	0.625	15	0.800	0.380	0.253	0.802
	DMFT	8	0.375	0.375	15	0.667	0.303	0.585	0.565
Obese	DS	6	0.833	0.833	6	0.833	0.833	0.000	1.000
	MS	6	0.000	0.000	6	0.000	0.000	. . . .	. . . .
	FS	6	0.000	0.000	6	0.000	0.000	. . . .	. . . .
	DMFS	6	0.833	0.833	6	0.833	0.833	0.000	1.000
	DMFT	6	0.667	0.667	6	0.667	0.667	0.000	1.000

D. decayed, M. missing, F. filled, T. teeth, S. surfaces

Table 3 shows the differences in the severity of permanent teeth caries between anxiety groups. There was a higher mean of D1 (initial enamel caries) in non-anxious than anxious children, with a statistically significant difference (p<0.05) only in normal-weight children. The differences in the means of D2, D3, and D4 between anxiety groups were not significant. Not all children in the study had erupted permanent teeth, resulting in 71 children in Table 2 and Table 3.

**Table 3:** Severity of permanent teeth caries across anxiety groups based on nutritional status

Nutritional status	Caries severity	Anxiety groups						T value	p-value
		Anxious			Non-anxious				
		N	Mean	±SE	N	Mean	±SE		
Normal weight	D1	16	0.188	0.136	20	0.850	0.233	2.459	0.020*
	D2	16	0.313	0.120	20	0.800	0.329	1.391	0.177
	D3	16	0.188	0.136	20	0.150	0.082	0.236	0.815
	D4	16	0.000	0.000	20	0.000	0.000	. . . .	. . . .
Overweight	D1	8	0.250	0.164	15	0.867	0.376	1.503	0.150
	D2	8	0.625	0.625	15	0.667	0.347	0.058	0.955
	D3	8	0.000	0.000	15	0.000	0.000	. . . .	. . . .
	D4	8	0.000	0.000	15	0.000	0.000	. . . .	. . . .
Obese	D1	6	0.167	0.167	6	0.000	0.000	1.000	0.363
	D2	6	0.833	0.833	6	0.833	0.833	0.000	1.000
	D3	6	0.000	0.000	6	0.000	0.000	. . . .	. . . .
	D4	6	0.000	0.000	6	0.000	0.000	. . . .	. . . .

* Significant difference (p<0.05), D1: initial caries, D2 enamel caries, D3 dentine caries, and D4 pulp involvement

**Figure 1:**
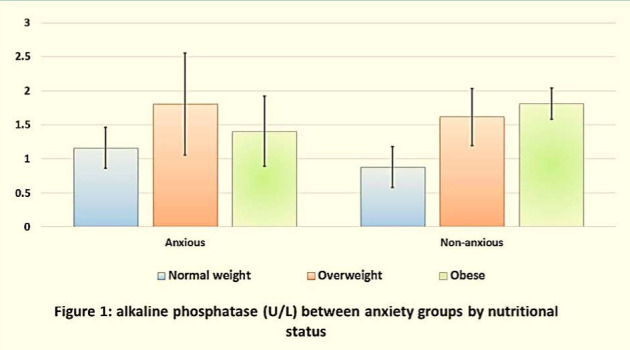
Alkaline phosphatase levels (U/L) between anxiety groups based on nutritional status

Table 4 presents the prevalence of dental caries in primary teeth between anxiety groups according to nutritional status. No statistically significant differences between anxious and non-anxious children were observed in the mean values of dmfs, dmft, and their components. Additionally, there were no statistically significant differences in the severity of caries in primary teeth between anxiety groups based on nutritional status, as shown in Table 5. Regarding the levels of alkaline phosphatase, various means were found, but no statistical differences (p>0.05) in AlP were observed between non-anxious children and anxious children in each nutritional status group, as depicted in Figure 1.

**Table 4:** Primary teeth caries between anxiety groups based on nutritional status

Nutritional status	Dental caries	Anxiety groups						T value	p-value
		Anxious			Non-anxious				
		N	Mean	±SE	N	Mean	±SE		
Normal weight	DS	21	14.000	2.802	20	12.700	1.573	0.399	0.692
	MS	21	0.000	0.000	20	0.000	0.000	. . . .	. . . .
	FS	21	0.000	0.000	20	0.000	0.000	. . . .	. . . .
	DMFS	21	14.238	2.812	20	13.700	1.608	0.164	0.871
	DMFT	21	6.571	1.001	20	6.200	0.501	0.327	0.746
Overweight	DS	11	10.636	2.861	15	13.000	2.418	0.632	0.533
	MS	11	0.000	0.000	15	0.000	0.000	. . . .	. . . .
	FS	11	0.000	0.000	15	0.000	0.000	. . . .	. . . .
	DMFS	11	10.636	2.861	15	13.667	2.431	0.808	0.427
	DMFT	11	4.818	1.182	15	6.000	0.976	0.776	0.446
Obese	DS	8	10.875	1.922	7	9.286	2.190	0.548	0.593
	MS	8	0.000	0.000	7	0.000	0.000	. . . .	. . . .
	FS	8	0.000	0.000	7	0.000	0.000	. . . .	. . . .
	DMFS	8	11.500	1.637	7	9.286	2.190	0.823	0.425
	DMFT	8	6.750	0.996	7	4.714	0.969	1.456	0.169

D. decayed, M. missing, F. filled, T. teeth, S. surfaces

**Table 5:** Severity of primary teeth caries between anxiety groups based on nutritional status

Nutritional status	Caries severity	Anxiety groups						T value	p-value
		Anxious			Non-anxious				
		N	Mean	±SE	N	Mean	±SE		
Normal weight	d1	21	0.571	0.245	20	0.750	0.204	0.558	0.580
	d2	21	1.429	0.406	20	1.300	0.341	0.241	0.811
	d3	21	11.238	2.476	20	8.850	1.539	0.810	0.423
	d4	21	1.810	0.745	20	2.550	0.806	0.675	0.503
overweight	d1	11	0.545	0.247	15	0.733	0.284	0.477	0.638
	d2	11	1.909	0.680	15	1.133	0.307	1.139	0.266
	d3	11	8.273	2.494	15	9.800	2.245	0.452	0.656
	d4	11	0.455	0.455	15	2.000	0.822	1.486	0.150
Obese	d1	8	1.000	0.866	7	1.286	0.969	0.221	0.829
	d2	8	3.000	0.982	7	1.857	0.705	0.920	0.374
	d3	8	7.875	1.913	7	7.429	2.034	0.160	0.875
	d4	8	0.000	0.000	7	0.000	0.000	. . . .	. . . .

d1: initial caries, d2: enamel caries, d3: dentine caries, d4: pulp involvement

## DISCUSSION

Dental anxiety remains a significant and persistent issue that could influence the utilization of dental services. Various studies have highlighted the use of physiological measurements, such as oxygen saturation and heart rate, to assess dental anxiety, with the finger oximeter chosen in this study due to its direct and noninvasive nature.

In our study, elevated heart rates in school-aged children were considered suggestive of anxiety, consistent with findings from previous research [16]. On the other hand, no significant difference in oxygen saturation between the two groups was reported, which agrees with the study of Tshiswaka and Pinheiro [17]. The level of oxygen saturation can change in relation to other factors like age, gender, and blood pressure. While some earlier studies have reported associations between dental anxiety and dental caries prevalence [3, 4, 18], our research did not find such relationships, both in primary and permanent teeth. The differences in methodologies, age groups, method of data analysis, and the methods used in dental anxiety assessment are factors that could affect the study results.

In addition to that, dental caries is a chronic condition influenced by many factors. Due to the impact of children's nutritional status on their health and considering previous evidence linking nutritional status to dental caries [7 ,8], our study aimed to explore the relationship between dental caries and anxiety within the context of children's nutritional status. Non-anxious children with normal weight status had higher mean values of initial caries, and this difference was statistically significant. It is possible that non-anxious children and parents could be less aware of oral hygiene practices and regular dental visits. This finding disagrees with the results of DeDonno, who concluded that dental anxiety was correlated with poor oral hygiene practice [19]. Anxious children might spend more time brushing their teeth to avoid dental visits.

Previous studies have explored associations between dental anxiety and salivary enzymes, such as cortisol and amylase [20, 21]. We hypothesized that dental anxiety might also impact salivary alkaline phosphatase, which could subsequently affect dental caries. However, our results showed no significant differences in the levels of this enzyme between anxious and non-anxious children. This indicates that salivary alkaline phosphatase may not be directly influenced by dental anxiety, and its levels may be influenced by other factors.

Longitudinal studies investigating anxiety, salivary biomarkers, and dental caries are essential to gain deeper insights into the complex nature of this disease and to develop novel approaches for its management. Some studies have indicated a positive correlation between salivary AlP and dental caries [22, 23], while others found no impact of nutritional status on salivary alkaline phosphatase levels [24]. Alkaline phosphate level was decreased in malnourished children [25]. This controversy in the relationship between the enzyme and weight status led the authors to explore this hypothesis further based on the child's nutritional status. One of the limitations of this study is the wide age range, which may introduce variability, as dental anxiety levels can differ among different age groups. Additionally, the nutritional status groups were not equal in size, which can be challenging to avoid given the natural variations in heights and weights among children.

## CONCLUSION

Dental anxiety had no effects on the prevalence of caries in primary teeth across different nutritional statuses. Furthermore, dental anxiety did not have any significant effects on dental caries in overweight and obese children. However, dental anxiety had an effect on dental caries in permanent teeth among children with normal weight, where it appeared to reduce the occurrence of initial enamel lesions in anxious children. There was no significant relationship between dental anxiety and the level of salivary alkaline phosphatase in children with different nutritional statuses.

## Data Availability

Further data is available from the corresponding author upon reasonable request.

## Personal thanks

We want to thank Asst. Prof. Dr. Bushra Ni’ma Rashid for linguistic assessment.
